# Permanent draft genome sequences of cadmium-resistant isolates of *Cupriavidus* from soils within the Tar Creek Superfund site

**DOI:** 10.1128/mra.00818-24

**Published:** 2024-11-26

**Authors:** Jordon N. Winn, Emily A. Wedlock, Emily P. McHugh, David Monismith, Jr., James H. Campbell, Alisha G. Campbell

**Affiliations:** 1 Department of Natural Sciences, Northwest Missouri State University, Maryville, Missouri, USA; 2 Independent Researcher, Oklahoma City, Oklahoma, USA; 3 Loess Hills Research Center, Department of Natural Sciences, Northwest Missouri State University, Maryville, Missouri, USA; University of Southern California, Los Angeles, California, USA

**Keywords:** cadmium, lead, zinc, cobalt, *Cupriavidus*, czcCBA, Tar Creek Superfund site

## Abstract

Soil samples taken near the abandoned town of Picher, OK, USA, were used to enrich and isolate bacteria in the presence of cadmium. Isolates reported belong to the genus *Cupriavidus*. Here, we report their permanent draft sequences with an emphasis on genes conferring resistance to cadmium.

## ANNOUNCEMENT

The Tar Creek Superfund site is found within the larger Tri-State Mining district along the Oklahoma, Kansas, and Missouri borders (1). Lead mining was active within this area from 1904 to 1970 and left more than 68 million metric tons of waste (1). This waste contains elevated levels of Pb, Zn, and Cd (2) that continue to impact the microbial ecology of this area (3). Our sequenced isolates from soils at this site give insight into survival mechanisms used in the presence of elevated levels of heavy metals, such as Cd.

Topsoil samples were collected from the Tar Creek Superfund site. Sample locations and collection methods are described in Beattie et al. (2). One mL of a 10^−1^ dilution (1 g of soil in 9 mL of sterile dH_2_O) of each sample was used to inoculate 100 mL enrichment cultures in 0.5 × Nutrient Broth amended with either 20 or 150 ppm CdCl_2_·2.5 H_2_O (Table 1), and these were incubated at 30°C for 7 days. Aliquots (100 µL) were used to inoculate secondary enrichments in the same medium and growth conditions. Secondary enrichments were plated on solid media of the same composition at a 10^−6^ dilution and incubated under the same conditions. Isolated colonies were streaked three times for purity, and colony PCRs were used to amplify 16S rRNA genes (4) to identify isolates of interest.

**TABLE 1:** Genome-assembly statistics and cadmium-resistance gene annotations

	Isolate					
Genome characteristic	EM05	EM19	EM20	EM22	EM23	EM36
BioSample accession #	SAMN36938092	SAMN36938093	SAMN36938094	SAMN36938095	SAMN36938096	SAMN36938097
SRA #	SRS18576999	SRR25603816	SRS18577001	SRS18577002	SRS18577003	SRS18577004
GenBank genome accession #	JBEFLN000000000	JBEFLM000000000	JBEFLL000000000	JBEFLK000000000	JBEFLJ000000000	JBEFLI000000000
IMG accession #	2816332647	2816332648	2816332649	2816332611	2816332612	2816332614
Sample latitutude	36.9871	36.9871	36.9871	36.9871	36.9871	36.9871
Sample longitude	−94.7882	−94.7882	−94.7882	−94.8937	−94.8937	−94.7882
[Cd] in enrichment (ppm)	150	150	150	20	20	20
Assembler, *k*-mer used	IDBA-UD, 97	SPAdes, 99	SPAdes, 99	SPAdes, 99	SPAdes, 99	SPAdes, 99
Number of total reads	269,821	824,214	3,083,073	1,164,307	1,517,834	1,606,976
Assembly size (nt)	6,622,294	6,772,639	6,777,180	6,456,536	6,457,878	7,221,874
Contigs	923	132	78	122	121	182
Completeness (%)^*a*^	98.18	99.71	99.71	99.67	99.67	99.94
Contamination (%)^*a*^	1.56	1.15	1.15	0	0	0.15
GC content (%)	65.52	65.57	65.58	67.47	67.47	66.1
N50	10,407	91,443	179,020	94,257	111,071	78,683
L50	196	24	14	21	17	31
Total # genes	6,622	6,226	6,209	5,898	5,896	6,692
Protein-coding genes	6,559	6,157	6,140	5,802	5,802	6,600
RNA genes	63	69	69	96	94	92
Cd export genes *czcCBA*	3	4	4	4	5	5
Cd export gene *czcD*	2	2	2	2	2	2
Cd export gene *czcE*	1	1	1	1	1	2
Cd^2+^/Zn^2+^-exporting ATPase	1	3	3	1	1	3

^*a*^ Estimated by CheckM (v 1.0.13).

Cells were grown for 2 days in the same broth used for enrichment and isolation, pelleted at 5,000 × *g* and shipped to The Sequencing Center (Ft. Collins, CO, USA) for DNA extraction (Zymo Quick-DNA Fungal/Bacterial kit), library preparation (Nextera XT kit), and Illumina sequencing (MiniSeq High Output, 2 × 150 bp) following manufacturer’s protocols. Low-quality reads were removed from R1 and R2 files of each genome using BBTools (v38.32; sourceforge.net/projects/bbmap/) bbduk routine. Reads were assembled using IDBA-UD (v1.1.1) (5) and SPAdes (v3.10.1) (6) with a parameter sweep of *k*-mer values ranging from 25 to 99 in increments of 2. Assemblies were assessed using Quast (v5.0.1) (7), and optimal assemblies were selected based upon N50 values (Table 1). Afterward, contigs shorter than 1 kb were manually removed from the assembly. BLAST+ (v2.7.1+) (8) was used to screen for human contamination (human genome v38). Genome completeness and contamination (Table 1) were assessed using CheckM (v1.0.13) (9). Final assemblies were submitted to the Integrated Microbial Genomics database (10) for annotation and functional prediction (Table 1). Default parameters were used for all software unless otherwise specified.

**Fig 1:**
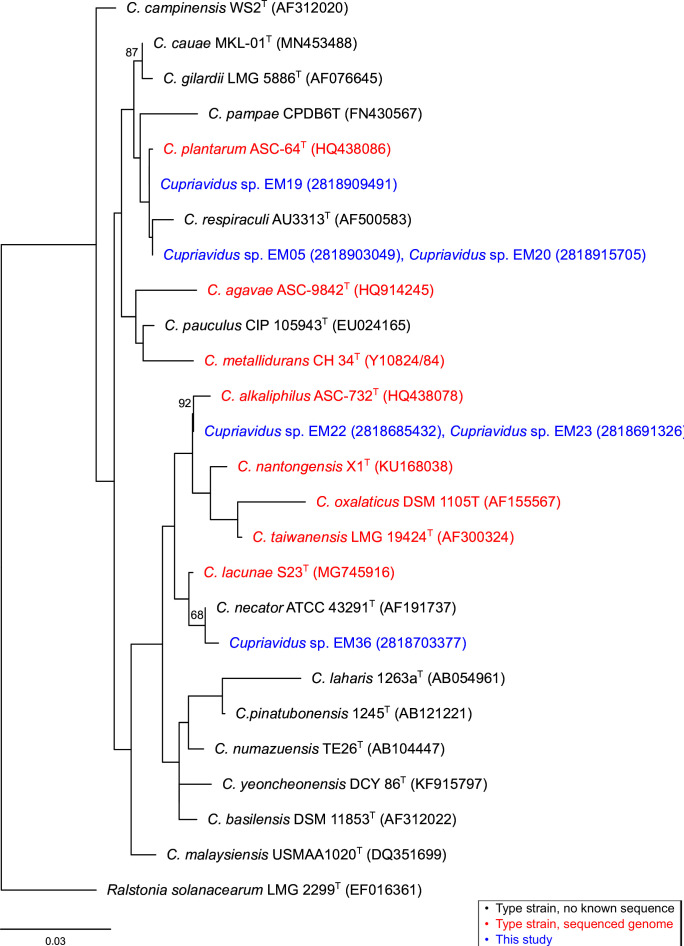
Maximum likelihood phylogeny of 16S rRNA genes. Mega11 (17) was used to align sequences using MUSCLE (18) and identify GTRGAMMA+I as the optimal substitution model. The phylogeny was created in RAxML (11) with 1,000 bootstrap replicates. All validly named species of *Cupriavidus* were included. Sequenced genomes are shown in green text, type strains without genome sequences are shown in black text and our strains are depicted in blue text. NCBI (alphanumeric) or IMG accession numbers (numeric) are listed in parentheses. When our strains’ sequences were found to be identical to one another (e.g., EM19 and EM23), one was removed from the alignment for construction of the phylogeny, as suggested by RAxML documentation (11). Subsequently, these names were added to the tree during annotation. Bootstrap support below 65% was omitted from the tree. Scale bar represents the nucleotide substitutions per site.

Optimal assemblies were estimated to range between 98–99% complete with 0–1.5% contamination. A maximum-likelihood phylogeny of 16S rRNA genes (Fig. 1) created in RAxML (v8.2.11) (11) indicated that our strains were most closely related to type strains *C. necator*, *C. alkaliphilus*, *C. respiraculi*, and *C. plantarum*. Our isolates encode several genes (Table 1) experimentally determined to be important for growth in the presence of high Cd concentrations (12–16), and analyses suggest Cd resistance is prevalent and similarly encoded across this genus.

## Data Availability

Illumina sequencing files have been deposited in the SRA under BioProject PRJNA1004322. Accession numbers for BioSamples, SRA, Genbank records, and IMG annotations are listed in Table 1.
